# Investigating Person‐Centred Care Planning in Care Homes Across England: An Exploratory Study of Practices and Contextual Factors

**DOI:** 10.1111/jan.16965

**Published:** 2025-04-07

**Authors:** Jacqueline Damant, Yuri Hamashima, Madalina Toma, Nick Smith, Jonathan Taylor, Thais Caprioli, Sarah Jasim, Laura Prato, Hugh Mcleod, Clarissa Giebel, Michele Peters, Anna Ferguson Montague, Lynne Wright, Martin Knapp, Anne‐Marie Towers

**Affiliations:** ^1^ Care Policy and Evaluation Centre London School of Economics and Political Science London UK; ^2^ Bristol Medical School University of Bristol Bristol UK; ^3^ Personal Social Services Research Unit University of Kent Canterbury UK; ^4^ Nuffield Department of Public Health University of Oxford Oxford UK; ^5^ Institute of Population Health University of Liverpool Liverpool UK; ^6^ Independent Patient, Carer and Public Involvement and Engagement advisors; ^7^ The Policy Institute King's College London London UK

**Keywords:** ageing, dementia, end of life, holistic care, long‐term care, nursing home care, older people, policy, qualitative approaches, residential facilities

## Abstract

**Aims:**

To report how person‐centred care principles are applied to care planning and to explore the contextual factors affecting their implementation in older adult care homes in England.

**Design:**

A combined framework analysis and quantitative content analysis study.

**Methods:**

Using a semi‐structured questionnaire, we interviewed 22 care home managers in England, exploring topics around care planning processes. Audio recordings were transcribed verbatim. Transcripts were analysed through a combined framework approach and content analysis.

**Results:**

Most care home managers discussed person‐centred care planning in terms of understanding residents' values and preferences and their engagement in decision‐making. Factors facilitating person‐centred planning implementation included accessible planning tools, supportive care home leadership, effective communication and collaborative partnerships. Inhibiting factors included regulatory and care practice misalignment, time constraints and adverse staffing conditions.

**Conclusion:**

Differences between care home practitioners' understanding and practice of person‐centred care planning require further examination to improve understanding of the sector's complexity and to develop suitable care planning instruments.

**Implications for the Profession:**

Findings demonstrate a need for improved staff access to specialised person‐centred care training and an opportunity for care home nursing practitioners to lead the co‐development of digital person‐centred care planning tools that reflect the reality of long‐term care settings.

**Impact:**

Identifying factors influencing the implementation of holistic approaches to care planning makes clear the need for modernising long‐term care policy and practice to adapt to the contemporary challenges of the care home sector.

**Reporting Method:**

Study reporting was guided by the Standards for Reporting Qualitative Research.

**Patient or Public Contribution:**

Two public involvement advisors with lived experience of caring for a relative living in a care home contributed to the development of the interview guide, advised on care home engagement, guided the interpretation of the findings and commented on the drafted manuscript.

**Summary**

- Identification of the extent to which person‐centred care principles are incorporated in care planning practice, and the factors that influence its implementation, highlight areas for improvement in care home nursing practice and education. This can serve to instigate nurse practitioners' to lead the redesign of holistic care planning tools that will facilitate communication and continuity of care across services.
- The findings can also inform health and social care policies endeavouring to modernise service delivery models and integrate care systems that are responsive to the imbalances of care demands and availability of care resources in ageing populations.

## 1 Introduction

Care homes in England currently support around 300,000 people aged over 65 (Office for National Statistics 2023), 70% of whom are living with dementia (Berg 2025). A high proportion of older care home residents have multiple morbidities and degenerative conditions and require extensive support for activities of daily living (ADLs), including eating, washing and dressing and support for engaging in social and leisure events (Farrington 2014). The care home market in England is characterised as a mixed economy of residential and nursing care home providers from private, local authority, not‐for‐profit and National Health Service sectors. Over 80% of all care home beds are provided by the private, for‐profit sector (Social Care Institute for Excellence 2025).

Care planning is a vital part of ensuring that care home services consider residents' evolving needs by setting out the type, frequency and level of support required. Care plans' contents are determined by individual service providers. Typically, care plans contain scheduling and resource requirements related to residents' nutrition, personal care, medication and mobility needs. A person‐centred care (PCC) approach to care planning is a collaborative process, actively involving the person with care needs, those close to them and care staff, with the aim of designing a comprehensive, personalised support plan that reflects the person's preferences, needs and aspirations (Forsgren and Saldert 2022). Person‐centred care planning (PCCP) has been promoted across a range of health and social care settings worldwide, including within residential care (Lepore et al. 2018; Smith et al. 2024; Bennett et al. 2020; Abbott et al. 2016). However, despite the impetus for adopting PCCP approaches, little is known about whether current care planning practices in older adult residential care settings reflect PCC concepts (Lepore et al. 2018; Sussman et al. 2023; Steel et al. 2022). Therefore, to enhance understanding of long‐term care planning for older adults, this paper examines how PCC principles are applied in English care homes and the contextual factors influencing their implementation.

## 2 Background

PCC aims to actively engage people who draw on care services—or their advocates—in making decisions about how to address their unique needs, values and preferences by fostering trusting relationships amongst persons using care, care practitioners and key members of the persons' family networks (Forsgren and Saldert 2022; Coulourides Kogan et al. 2016; Manthorpe and Samsi 2016). PCC has evolved to be recognised as the ‘gold standard’ of care for meeting the varied needs of ageing populations (Backman et al. 2021). In 2001, the UK Government's Department of Health and Social Care published the *National Service Framework for Older People*., which marked an initial policy shift away from standardised service‐led care towards personalised approaches, by stipulating that older service users be treated as individuals and care packages reflect their personal needs, in all care settings (Department of Health and Social Care 2001). Subsequent guidance from the *National Institute for Health and Care Excellence* and *Social Care Institute for Excellence* (SCIE) reinforced the government's personalisation agenda, by encouraging people's involvement in decisions about their care, and for care services to be responsive to people's identity and preferences (National Institue for Health and Care Excellence 2015a, 2015b; Social Care Institute for Excellence 2017). These themes were re‐emphasised in the *Putting People at the Heart of Care* white paper (2021) (Department of Health and Social Care 2021), which acknowledged the need for further public investment to support the delivery of timely and holistic PCC. The Care Quality Commission (CQC), the care service regulator in England, published a new regulatory strategy that also embraced a ‘collaborative approach’ to ensure that care recipients are ‘able to influence the planning of and…[are] truly involved as equal partners in their care at all levels’ (Care Quality Commission 2020, 15). Such policies received broad empirical support, demonstrating the benefits of PCC interventions across health and social care settings (Blake et al. 2020). Strong evidence links the adoption of PCC attitudes and approaches in care homes to improved resident behaviours and wellbeing, reduced use of psychotropic medications and greater job satisfaction (Backman et al. 2021; Brown Wilson et al. 2013; Gustavsson et al. 2023; van den Pol‐Grevelink et al. 2012; Ballard et al. 2018; Erkes et al. 2022).

The UK care policy also specifically advocates for the adoption of person‐centred care approaches in *care planning* processes, across care settings. The *Care Act 2014* requires local authorities to provide PCC at every stage of the assessment, planning and delivery of care, ‘regardless of the care setting where needs are met’ (Department of Health and Social Care 2014) (p.168). Additionally, the CQC requires all registered providers to adhere to care planning and delivery practices that fully reflect people's holistic needs and preferences and encourages people's engagement in decision‐making about their care and treatment (Care Quality Commission 2024a). Nevertheless, little evidence exists about the adoption of PCCP practices, and the factors influencing their application remain largely unexplored. Most research exploring PCCP practices in residential care homes relates to end‐of‐life (EOL) and advance care planning (ACP). This work has primarily explored the effects of PCC ACP training on care home staff self‐efficacy (Baron et al. 2015; Coleman et al. 2017; Dobie et al. 2016; Kesten et al. 2022; O'Brien et al. 2016; Spacey et al. 2021; Stone et al. 2013; Van den Block et al. 2019), and the impacts of implementing PCC ACP on hospitalisation rates (Bavelaar et al. 2023; Finucane et al. 2013; Garden et al. 2022; Sopcheck and Tappen 2023; Wickson‐Griffiths et al. 2014) and on the wellbeing of residents and family carers (Bavelaar et al. 2023; Wickson‐Griffiths et al. 2014). The literature on general PCCP practices in care homes is more limited. Forsgren and Saldert (2022), Brown Wilson et al. (2013) and Kang et al. (2020) have examined resident‐staff relationships and communication in the context of residents' engagement in care planning and found that different approaches influence residents' sense of purpose and belonging. Backman et al. (2020) found that care home managers believed that PCCP is fundamental to delivering meaningful support to older residents. A recent study of care planning practices, however, found that although care home practitioners valued PCC approaches, there were few indications that care planning was being conducted in a person‐centred way (Smith et al. 2024). Likewise, Abbott et al. (2016) noted that care home staff generally supported the incorporation of residents' preferences in care planning but often felt inhibited by the facility's social environment and adherence to task‐oriented care practices.

## 3 The Study

This study aims to further the understanding of current PCCP practices and the contextual factors affecting their implementation in care homes for older people in England. To address this aim, we set out to answer two research questions (RQ):

RQ1. To what extent is care planning in care homes conducted in a person‐centred way?

RQ2. What barriers and facilitators influence the implementation of PCCP in care homes?

## 4 Methods

### 4.1 Study Design and Recruitment

This study, which combines a qualitative framework—and quantitative content—analysis, is part of the Well‐being in Care Homes research project, supported by the National Institute for Health Research (NIHR) Applied Research Collaboration (ARC) Kent, Surrey and Sussex (NIHR Applied Research Collaboration Kent SaS 2020). For details about the broader NIHR ARC programme, please see Appendix S1. A detailed description of the methods of the study is described elsewhere (Smith et al. 2024). Social care professionals involved in the care planning process in older adult care homes were purposively recruited to participate in a semi‐structured interview. The study was advertised via the NIHR ARCs, NIHR local clinical research networks, academic health science networks, Enabling Research In Care Home (ENRICH) and the Contact, Help, Advice and Information Network (CHAIN). The research team also drew on existing contacts and social media platforms such as Facebook and X.

## 5 Patient, Carer and Public Involvement and Engagement (PCPIE) Statement

Two PCPIE advisors, with experience of caring for a relative living in a care home, contributed to the development of the interview guide. They also provided advice on engaging with care homes, as well as comments and suggestions regarding the interpretation of the findings and the final paper.

### 5.1 Ethical Considerations

Ethical approval for this study was granted by the Staff Review Committee, Division for the Study of Law, Society and Social Justice, The University of Kent on 19/07/2022 (application ref. 692).

### 5.2 Data Collection

Between September and December 2022, 6 researchers (NS, SJ, JT, HM, JD, LP) conducted 21 semi‐structured interviews with 22 care home practitioners. The interview topic guide (Appendix S2) was shaped by findings from a scoping review of care planning (Taylor et al. 2023) and included themes around the aims of care planning; current care planning processes; challenges to conducting care planning, and improving care planning (Smith et al. 2024). While PCCP was not explicitly addressed in the topic guide, PCC was an underlying concept of the study inquiry.

Participants were provided with the option of either a face‐to‐face or remote interview, facilitated through videoconferencing platforms (e.g., Microsoft Teams or Zoom). One interview (NT04) was attended by a single participant and two researchers. Another interview (NWC0102) was held by a single researcher and two participants from the same organisation with different roles in care planning. The remaining interviews adhered to a one‐to‐one structure. Further details of data collection methods are described in Smith et al. (2024). Informed consent was obtained from all participants prior to data collection.

Interviews, lasting an average of 36 min (range 25–60 min) were audio‐recorded and transcribed verbatim by a professional transcriber, ensuring the removal of any identifying information.

### 5.3 Theoretical Frameworks and Data Analyses

#### 5.3.1 RQ1. Application of PCC Principles in Planning

There are several PCC assessment tools designed for primary data collection in various care settings (de Silva 2014). However, the interview topic guide for the current study covered issues relating to general care planning, without overtly addressing PCCP. Therefore, in a secondary analysis of the interview data, we deductively coded reported practices based on Wilberforce et al. (2017) thematic framework. This framework synthesises care practices associated with PCC and helps to clarify the often ambiguously defined concept of person‐centredness by highlighting its core components (see Box 1).

To assess the extent to which care planning is conducted in a person‐centred way, the transcripts were analysed through three overarching dimensions of PCC proposed by Wilberforce et al. (2017): (1) understanding the person, (2) engagement in decision‐making and (3) promoting the care relationship, with each dimension comprising four constituent constructs (see Box 1).

The interviews were analysed in Nvivo (version R1) using a framework approach according to the principles outlined by Ritchie and Lewis (2003), supplemented by a quantified content analysis set out by Bengtsson (2016). A combined quantification–qualitative approach enhances the thematic analysis by illustrating the degree to which different aspects of PCC were addressed in the consultations (Bengtsson 2016).

In the first stage, transcripts were read through by seven researchers (JD, YH, SJ, LP, NS, JT and MT) to familiarise themselves with the data. In stage two, open coding techniques were applied, where researchers inductively generated codes around themes related to person‐centred care. During the third stage, three researchers (JD, YH and MT) deductively grouped the codes into themes related to the dimensions and constructs of the Wilberforce et al. (2017) framework and developed a refined analytical framework. In phase four, seven researchers (JD, YH, SJ, LP, NS, JT and MT) re‐coded the transcripts by applying the refined framework and charted the re‐coded interview excerpts into a framework matrix. In the final phase, three researchers (JD, YH and MT) met to resolve disagreements and synthesise the findings.

Next, JD conducted the content analysis in Microsoft Excel, by counting the number of participants and the proportion of the total sample (*n* = 22), who discussed each unit of analyses: the three domains and the twelve individual domain constructs of the framework proposed by Wilberforce et al. (2017). The two Interviewees participating in interview NWC0102 were counted as two individual participants. A participant discussing an individual PCC construct one or more times during the interview was counted once towards the number of participants discussing the respective construct. A participant discussing one of the four constructs within a domain one or more times during the interview was counted once towards the number of participants discussing the relevant domain. Participants discussing more than one construct within a domain were counted only once towards the number of participants discussing the respective domain.

#### 5.3.2 RQ2. Factors Influencing the Implementation of PCCP

To answer RQ2, identifying the factors that influence the implementation of PCCP in care homes, we engaged in the identical five‐phased framework approach described above, using a version of the Consolidated Framework for Implementation Research (CFIR) (Damschroder et al. 2022) that was modified for the current study. As a well‐established meta‐theoretical framework to promote effective implementation, the CFIR was chosen for its capacity to comprehensively analyse what works, where and why across multiple contexts. Resulting CFIR analyses allow for an overview of the multi‐level determinants influencing implementation outcomes and enable comparisons between similar settings (Damschroder et al. 2009).

**FIGURE 1:**
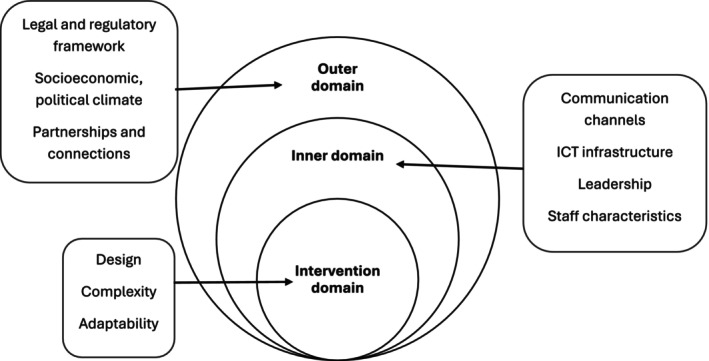
Domains and constructs of an adapted CFIR model. *Source*: Damschroder et al., 2022.

The modified CFIR framework considered the contextual determinants (i.e., barriers and facilitators) to implementing PCCP according to three domains: the intervention domain, the inner setting and the outer setting domains. A description of the domains and their respective constructs in the context of PCCP is illustrated in Figure 1. The original CFIR framework includes the fourth and fifth domains: individual and implementation process, respectively. The individual domain is combined here with the inner setting domain, where the qualities of staff and the leadership are discussed in the context of the internal care home environment. Data were not collected around topics related to strategies and mechanisms for implementation; therefore, the implementation process domain does not form part of the current version of the CFIR framework.

## 6 Findings

### 6.1 Participant Characteristics

Of the 22 care home practitioners taking part in the study, the majority were female (81.8%), of white ethnicity (86.4%), aged between 45 and 64 (51.9%) and held a leadership role within the care home (54.5%) (see Table 1). Several of the participants with registered manager and other leadership roles mentioned having a background in adult nursing.

**TABLE 1:** Participant characteristics.

	Total (%), *N* = 22
Gender	
Female	81.8
Male	18.2
Age	
25–44 years	40.9
45–64 years	59.1
Ethnicity	
Asian/Asian British	4.5
Black/Black British	4.5
Mixed/Multiple	4.5
White	86.4
Job Role	
Activity staff	4.5
Medical Doctor	4.5
Registered manager	13.6
Registered nurse	22.7
Other leadership roles^a^	54.5

^a^ Other leadership roles included Deputy Care Home Manager, Care Quality Manager and Head of Care.

### 6.2 Application of PCC Principles in Care Planning: Quantitative Content Analysis

Results of the content analysis, displayed in Table 2, show that more than 80% of participants referred to at least one construct relating to *understanding the person* (86.4%) and *engagement in decision‐making* domains (81.8%) Half of the participants discussed at least one construct of the *promoting care relationships* domain. Over 40% of participants discussed at least one construct of all three domains. Over a third of participants discussed two of the three PCC domains; the *understanding the person* and *engagement in decision‐making* domains were most frequently (27.3%) discussed together. Three participants discussed only one domain, two of which related to the *understanding the person* domain. One participant did not discuss any PCC domain in terms of the Wilberforce et al. framework.

**TABLE 2:** Number and percentage of participants addressing the PCC domains and constructs.

Dimensions/construct	*n* (% of Total *N*), Total *N* = 22
1. Understanding the person	19 (86.4)
Different dimensions of life requiring support	11 (50)
What is important to person's identity and wellbeing	9 (41)
Person's values and preferences in care	16 (73)
Personal experience of illness and limitations	1 (4.5)
2. Engagement in Decision‐making	18 (81.8)
Involvement in decision‐making process	12 (55)
Person's wishes shape care plans	11 (50)
Flexible care tailored to individual preferences	8 (36)
Information, options are clear	3 (14)
3. Promoting care relationships	11 (50)
Friendly, caring, respectful interactions	3 (14)
Positive attitude to person's capabilities	8 (36)
Continuity and coordination in care	7 (32)
Reciprocity	1 (4.5)
Summary count	
Three domains	9 (40.9)
Two domains only	8 (36.4)
Domains 1 and 2	6 (27.3)
Domains 1 and 3	1 (4.5)
Domains 2 and 3	1 (4.5)
One domain only	3 (13.6)
Domain 1	2 (9.1)
Domain 2	1 (4.5)
Domain 3	0 (0.0)
No domain	1 (4.5)

The most frequently discussed construct, understanding the person's values and preferences in care, was referenced by almost three quarters of participants (72.7%). The least discussed constructs, understanding personal experiences of illness and limitations and reciprocity in care relationships, were raised by one participant each in relation to care planning. Of the 12 PCC constructs, four were addressed by at least half of the participants.

### 6.3 Application of PCC Principles in Care Planning: PCC Framework Analysis

The following section presents examples of how care practitioners approached care planning in a person‐centred way, using the dimensions included in the Wilberforce et al. framework (Wilberforce et al. 2017).

#### 6.3.1 Understanding the Person

Wilberforce et al. (2017) describe the *understanding of the person* dimension as recognising the need to devise comprehensive, tailored responses to an individual's unique needs, aspirations and circumstances. As one participant expressed, this involves recognising the range of issues for which an individual requires support, beyond medical and ADL needs and ‘taking into account all of their needs, their choices, their beliefs, their dignity and history’, (KSS01). Half of the participants discussed the construct of knowing the different dimensions of life requiring support (Table 2), in terms of incorporating residents' emotional and social circumstances when developing care plans:

You have a care plan to identify the needs of the individual that you're going to be caring for and in the best way that their needs can be met by identifying what's important to them… it's not just about the health and safety aspect… it's a holistic approach, it's about meeting all of the needs, the spiritual as well as the physical and their emotional…. (KSS02)

Wilberforce et al. (2017) defined a second construct of the *understanding the person* domain as appreciating what is important to a person's identity, their interests, achievements and goals. This practice stems from the concept of identifying the ‘personhood’ of people living with dementia (Kitwood and Bredin 1992), who may be unable to verbally communicate their needs and wishes or explain their behaviours. Several participants explained using residents' life stories, past hobbies and interests to develop personalised care plans, often with help from ‘…the family to see if there's any extra information that they can provide to us’ (NT03):

Whatever they liked doing before, they should be able to carry on doing that and that might be pottering around in the garden, or it might be reading the paper or it might be doing bookies odds. They shouldn't be trammelled down into the bingo or whatever. (NWC0102)

Another construct of *understanding the person* is appreciating residents' personal values and preferences in care by identifying their priorities, their likes and dislikes of various forms of support (Wilberforce et al. 2017). Many participants mentioned the importance of incorporating residents' care goals in the care planning process. As one participant articulated, care planning should centre around residents' preferences, over and above others' priorities and opinions:

The relative might come to visit and say: ‘We want our mum up and dressed,’ but the mum might not want to be up and dressed…We have to follow what the resident wants. It's not what their relative wants. It's not what your colleague wants. It's not what the day staff want, that the night staff should wash everybody in the morning before they come in. It's what that person as an individual wants.(KSS04)

The final construct of the *understanding the person* domain is an understanding individuals' perceptions of their limitations and care needs (Wilberforce et al. 2017). This construct was raised by one participant. This could be because a large proportion of older care home residents live with significant cognitive impairment (National Institue for Health and Care Excellence 2024) and so may lack insight into their limitations or ‘may be unable to communicate’ (KSS01) their experiences of illness.

#### 6.3.2 Engagement in Decision‐Making

More than half of the participants suggested that involving residents, as much as possible, in care planning was a key aspect of the care home's person‐centred principles:

The key thing is the resident themselves. We make sure that the resident is driving the care plan essentially, because it is their document, it's just our role to say that they're updated regularly and that they're accurate. (WES02)

One participant demonstrated that residents' involvement in care planning was not constrained by cognitive and communication limitations. They described employing alternative methods to enable residents' preferences to inform their support, such as observing their behaviours and interpreting their body language:

…sometimes you can still see their habits in the things they want to do which can go into the plan, so it's always the resident first who will determine what goes in the plan by way of talking to them and seeing them. (KSS03)

Often in the context of ACP and EOL care, participants referred to the Wilberforce et al. (2017) construct of individuals' wishes and preferences shaping the development of care plans. In some cases (KSS04, NT02), the description of the ACP process was restricted to recording residents' options for resuscitation. Other participants, however, described more comprehensive approaches to EOL care planning, including the consideration of residents' personal religious rituals, preferred care setting and funeral arrangements:

Everybody fills [an ACP] when they come in, we treat it as part of the care plan … [The] ACP can be detailed: ‘Do you have a living will in place; Yes/No? Where is your preferred place of care at end of life? Any additional requests? Is there any active treatment you wish to have or wouldn't have?… Do you have any religious or cultural needs for them to describe what they want?… Is there any preferred funeral director that you would like to use?’ Then it goes down to specifics: ‘Is there anything specific [we] need to be aware of? Is there any music or people you want to be around you?’ (OTV02)

Wilberforce et al. (2017) also suggest that PCC should be adaptable and responsive to residents' individual decisions and preferences. Similarly, participants suggested that care plans should be ‘alive and flexible and real documents’ (KSS05) to reflect changes in residents' preferences, needs and goals:

[The] care plan needs to tell a story and like anybody else, you or me, this changes over time…Everything changes, things change all the time so it's always better to [revise] the care plans every day. (NWC0102)

Finally, to facilitate residents' engagement in care planning, Wilberforce et al. (2017) propose that a person‐centred approach should involve the use of appropriate communication methods and language so that people have a clear understanding of the support available. A small number of participants referred to techniques used to ensure residents' involvement in their care plans. This was achieved by providing transparent, accessible information for residents—and their families—so that they could make informed decisions about their care:

We give them as much information as we can on the organisation so that they can make an informed decision…so it's a bilateral agreement in our sort of minds… It also needs to be readable and understandable for the resident and their family themselves. (WES02)

#### 6.3.3 Promoting the Care Relationship

The third foundation block of Wilberforce et al. PCC framework is the interpersonal relationships formed between people using care services and care providers, based on mutual respect and trust, and appreciation of each other's contributions (Wilberforce et al. 2017). Participants described how attentive and caring engagement with residents facilitated the development of holistic care plans that would positively impact residents' wellbeing and quality of life:

[Care planning] is a great way of … really getting to know the person. That's what it's all about. It's sometimes not necessarily you capturing every little, tiny detail but it's that conversation: ‘Tell me about you. What have you done in your life? What are you most proud of? What have you achieved? What's important to you?’…And that's what makes me able, as a provider, to make sure that me and my team give you the kind of life here that you want to be able to live. (KSS05)

Promoting care relationships also entails recognising and encouraging residents' capabilities and goals, rather than focusing on their pathology and limitations (Wilberforce et al. 2017). Approximately a third of participants emphasised that adopting a strength‐based approach (Moyle et al. 2014) when developing care plans helped promote residents' independence:

[To] draw up a plan of action regarding their personal care…we would sit with the residents and say, ‘…how do you want us to help you meet these needs?’ We would draw up a document saying: ‘I prefer unperfumed products. I'd like a shower rather than a bath. Can you just wash my back for me? I can do everything else.’… whoever's delivering the care would be able to go: ‘I know exactly what that person wants me to do to help them remain independent’…. It is very much a person‐centred approach to the care plan… (NT02)

Promoting care relationships also relates to continuity and familiarity, resulting from a deep, personal understanding between residents and staff of each other's expectations (Wilberforce et al. 2017). Several participants commented that effective care planning facilitated the continuity of such understanding when engaging with external care organisations, professionals and agency staff. For instance, one participant noted that comprehensive care plans that describe residents' needs and preferences can be used to advocate for people who face difficulties speaking on their own behalf:

No matter which service you're there on behalf of, that care plan is your Bible. You should be able to go to it, reference it and actually use it in order to make sure that you're providing the absolute, right care as agreed with that person and their representatives. Because if that person can't talk for themselves or can't communicate with themselves, that care plan is king. (KSS05)

Finally, reciprocity refers to the mutual emotional benefits residents and staff gain from their care relationships (Wilberforce et al. 2017). The issue of reciprocity between care staff and residents in relation to care planning was rarely addressed during the interviews. One participant, however, outlined how a relationship‐centred model of care, adopted by the care home, influenced their approach to care planning:

We tried to take [care planning] away from the task…we focused a lot more on relationship‐centred care. The care plans were all about having relationships with the teams that look after you, who go the extra mile, the extra information outside of the care plan to deliver your care and to support you through the day. (KSS03)

### 6.4 Factors Affecting PCCP Implementation: CFIR Framework Analysis

Having identified examples of participants' application of person‐centred care in planning practice, the following section describes factors that participants felt supported and inhibited the implementation of PCCP in older adult care homes, using a modified version of the CFIR framework (Damschroder et al. 2022).

#### 6.4.1 PCCP Tools: Intervention Domain

The intervention domain considers how the design, complexity and adaptability of the care planning instruments themselves impact the implementation of PCC planning and delivery. Participants commented on the challenges of incorporating PCC principles in the planning process, particularly in terms of the time needed to continually collect and update care plans according to residents' changing preferences. It was also suggested that adopting PCC approaches to planning sometimes resulted in convoluted and inaccessible documents, which inhibited staff from absorbing essential resident information and, at times, detracted them from other essential care activities:

I personally think that care plans should be more practical… [but] it's all spieled out into some big flowery thing… [care staff] won't look at the care plan because it's too bulked up with nonsense… In fact, we're losing the actual main issue for [residents] in a whole spiel of things that we'd, obviously, do anyway for anybody… I'm under an enormous amount of strain from doing care plans and writing them. (KSS06)

Others suggested that some care planning tools failed to capture residents' personal preferences and values, which in turn negatively affected the delivery of PCC:

…a lot of the relationship‐centred preferences weren't necessarily documented…You would write the basics…There are lots and lots of problems with care planning. The actual tool itself…never met or provided the right questions or the right framework or the right level of integration to be able to deliver care…It's very one dimensional…. (KSS03)

Some participants suggested that PCCP tools should be accessible and adaptable to all care providers to facilitate the coordination and continuity of care. Participants explained that agency staff, for instance, should be able to ‘pick up the care plan and be able to look after somebody without having to ask anybody any questions’, (KSS01):

[The care plans] have to be detailed and concise enough that health professionals understand the detail of the individual, but readable and understandable enough so that if the resident or their family wishes to read it or see it or add anything, that they should be able to understand that themselves. (WES02)

#### 6.4.2 Care Homes: Inner Setting Domain

The inner setting domain of our modified CFIR framework combines the organisational characteristics of a facility or service where the intervention is implemented and staff characteristics. Some of the key determinants of the adapted inner domain include internal communication channels, the use of digital planning tools and the attributes of the care home leadership and staff.

Many participants observed that the way information was communicated across staff influenced the development of person‐centred planning. For instance, the continual exchange of resident updates at shift handovers, through informal staff conversations, online networks and ‘flash meetings…supervisions… [and key information] sent out as memos’ (KSS02) allowed all staff members to be abreast of individual residents' changing needs and preferences:

We all know some different thing [about residents]… The challenge is for everybody to know [the different aspects] so it is easily shared…We have handovers every morning, every night, if anything changes, we say [it] there. We [also] have a work WhatsApp… [For example, if] something quickly changes and I need everybody to know now, staff read those messages every day or before they come on duty. (NWC0102)

In relation to care homes' information communication technology (ICT) infrastructure, the introduction of digital care planning tools was a recurring theme amongst the interviews. Participants described the perceived benefits of digital care plans in relation to implementing PCCP, including updated care plans in real time, improved coordination of care across services, facilitated resident engagement in care planning and rapid access to residents' personal preferences and routines:

[The portable device] will say this person likes to have their breakfast at this time and they can be specific and say exactly what they want. Or it will say they need an aid, or they need assistance, or: ‘This is what time they like to get washed and dressed’… I can go on [to the system] and change [residents' care plan] and it will instantly upload. When the next person goes into that app it will tell them what's changed. (OTV02)

On the other hand, some participants expressed concerns that digital planning tools could detract from undertaking person‐centred approaches to planning:

We are in the process of implementing an electronic system which I don't really like that much because it contains different information and I can't really say—when it's not a free text for you to type—just some questions you need to answer. It's not that person centred and sometimes sentences come out a bit undignified… (OTV01)

The qualities of the care home leadership were also cited by participants (NT05, KSS03) as affecting the implementation of PCCPs. For instance, supportive care home managers who ‘lead by example’ (KSS02), are ‘visible on the floor’ (NT01) and ‘encourage [care staff] to bring ideas to the table’ (NWC04) enabled the development of care plans that reflected residents' wishes:

We're very lucky that we have the support of the management…If we go to them with an idea that a resident wants to be able to do this, then they will help us facilitate that, it's not a problem. (NT02)

One participant further explained how leadership lacking the appropriate skill set was a barrier to developing PCC approaches to care planning:

If the [registered manager] hasn't got the skills set, the experience, the determination [to take risks] then you've got no hope of implementing [person‐centred care planning]. (KSS05)

Several participants suggested that the implementation of PCCP required skilled staff. Some managers mentioned that many care staff lack appropriate levels of education, ‘are not confident with their writing skills’ (NT01) and ‘may not be highly literate’, creating ‘a lot of problems around documentation in care plans, extending through care planning in the home generally’ (KSS03). Several participants also suggested that providers who invested in developing staff PCC skills helped to promote effective, holistic care planning:

…for instance, in dementia, why is it that that symptom is being displayed? So how can you write a care plan when you don't quite understand the root cause of it? There is an obligation on providers to make sure that, at a very basic level, their staff are trained. Sometimes it's not about what support do you give them with the care plan, it's about what support do you do with the other things as well? (KSS05)

Staff attitudes towards care were also considered an important factor in PCCP. Motivation, compassion, confidence and an understanding of holistic care were described by participants as staff characteristics that were necessary to ensure that residents have ‘as much input as they want [into their care plans]’ (KSS02), which facilitated the implementation PCC planning and delivery:

I want [residents] to have the best life. I don't want them to sit in the chair all day, and you need to find staff which understands the same thing…you need the have the skill mix all the time and you need to have the right people [who] want this. (NWC0102)

Equally, staff lacking appropriate attitudes towards care were described as barriers to understanding the full range of residents' support needs (NCW0102) and were thought to be unable to contribute to a care team that fosters coordination and continuity in care planning:

…the quality of the [staff] we get…makes a massive difference to the way that they understand and care is delivered in a care home…there's a huge lack of emotional intelligence, in understanding what that is within a care home and understanding the needs of your other team members and other residents… (KSS03)

#### 6.4.3 Health and Social Care System: Outer Setting Domain

The outer setting domain captures the impact of external influences—such as national regulations and legal frameworks, socioeconomic and political climate and peripheral stakeholder and institutional partnerships—on an organisations' implementation plans (Damschroder et al. 2022). Recurring outer setting domain themes in the interviews related to working with external stakeholders, regulations and the current socioeconomic environment of the care home sector.

Many participants discussed co‐producing elements of care plans with external stakeholders, including family members and health and social care professionals. Incorporating external input and perspectives in the PCCP process allowed care homes ‘…to get information [from family members] about [the resident] as a person…[and] build a bigger picture’ (KSS06). Input from external stakeholders was credited with enabling care homes to develop care plans that reflect residents' interests, preferences, capabilities, while also recognising their limitations:

We worked with [the resident] and her care plan and we brought a few dementia community people in. We worked with external services and her GP and we talked to her. We're trying to build a picture…So that we can then go away and intervene appropriately. (KSS03)

It was also suggested that external input can pose challenges to the PCCP process. A few participants explained that sometimes, when relying on residents' families for additional information about their interests or EOL care wishes, they discovered family members had divergent priorities and opinions about residents' care:

[When deciding how] intensively to treat this person who is frail and elderly (at EOL), I have conversations with families …It's very time‐consuming because you ring, there's nobody there, or you find three siblings squabbling amongst each other for who is the leader…It's kind of messy, families. (NT05)

Regulations and standards were also raised as affecting the implementation of PCCP. Several participants commented that CQC guidelines helped to promote the development of holistic care plans:

[Care plans are] one of the key lines of enquiry that [the CQC] look at, [that] there is accurate care planning for the residents to meet their needs, [that] all the information is accessible, residents are involved and everyone is looking for a holistic approach…The inspector will come in and they will come and speak to a staff member and say: ‘Can you tell me about Mrs [name], what does she like, what does she not like?’ (OTV02)

Others, however, felt that ‘the sheer amount of guidance … you have to follow…and [the number of] external inspections you have’ (NT03) placed a barrier to implementing PCCP. Excessive regulations were blamed for the creation of care plans that simply ‘tick a box…to suit the CQC’ (KSS05) and where evidence beyond what is written down may not be meaningfully recognised by inspectors:

We have so many pictures everywhere of our residents doing things but do our care plans reflect their quality of life there? It's really difficult to evidence and that's why it's such a shame that nowadays our inspections from CQC are so focused on paperwork. If it's not written down it didn't happen. It's like: ‘Look around you, this is happening, it's just not written.’ (NT01)

Another dominant theme is related to staff shortages and high turnover rates. Many participants felt these issues contributed to strenuous workloads and affected the quality of PCC planning and delivery. One person commented ‘there's too much work for the amount of staff that is there’, (KSS04). High workloads were thought to be significant because implementing a PCCP approach required a considerable ‘use of resources, especially on staff and on time’ (KSS02). Another participant commented that:

One of the biggest challenges to care plans is time. If you look at it from a carer's perspective, [a lack of] time results in a lack of quality … [Q]uality is rarely delivered … because either the tools aren't there, the time isn't there, the staffing isn't there… (KSS03)

## 7 Discussion

This study explored the views and experiences of care home staff as they related to care planning practices within the current context of older adult care homes in England. Structuring our mixed analyses around Wilberforce et al. (2017) PCC and (modified) CFIR (Damschroder et al. 2022) frameworks enabled the contextualised exploration of how person‐centredness is applied to care planning practices and to gain an understanding of the barriers and facilitators that influence the implementation of PCCP in care homes.

### 7.1 Implications for Policy and Practice

Dissecting the application of the PCC concept into individual domains and constructs through a quantitative content analysis revealed a nuanced picture of care planning processes. Less than half of participants discussed PCCP in terms of all three of the Wilberforce et al. domains, and two‐thirds of the PCC constructs were discussed by a minority of participants, pointing to divergences not only in the interpretation of PCC but also in how care planning (in general) is implemented across different providers. The low frequency of discussing PCC in relation to planning could be indicative of the elusiveness of PCC as a concept and the lack of standardised PCC frameworks upon which care participants can base their practices (Coulourides Kogan et al. 2016; Manthorpe and Samsi 2016). Indeed, the study of the CFIR *inner setting* domain—which concerns care home managers' perceptions of the aptitude and motivation of some of the workforce –suggests that PCC principles are often misunderstood. This finding is consistent with reports of PCC‐related skills gaps across the sector (Abbott et al. 2016; Coleman et al. 2017; Bavelaar et al. 2023; Gilissen et al. 2021; Heckman et al. 2022). Emergent research also indicates a direct relationship between appropriate residential care staff training and effective PCC planning and delivery (Backman et al. 2021; Ballard et al. 2018; Fossey et al. 2014) and signals the challenges of addressing PCCP skills deficits in a sector with inordinately high staff turnover and where staff have limited access to appropriate training (Forsgren and Saldert 2022; Lepore et al. 2018; Kang et al. 2020; Stevens et al. 2022; Cooper et al. 2018; Guney et al. 2021). Furthermore, studies demonstrate persistent discrepancies between the understanding and practice of PCC principles amongst frontline care home staff, despite numerous policy and vocational initiatives promoting the resident and staff benefits of adopting PCC approaches (Cooper et al. 2018; Guney et al. 2021).

*Inner setting* findings further highlighted the positive impacts of supportive care home leadership, which clearly articulates PCC principles, and effective internal communication mechanisms on the incorporation of PCC approaches in resident care planning. Likewise, previous studies observed a positive association between PCC practice and care home leaders who promote the professional development of care staff and a supportive team environment (Abbott et al. 2016; Backman et al. 2021; Chenoweth et al. 2014). Developing the necessary skillsets has also been identified as a strategic component in policy efforts to transform the care sector into one that is receptive to the needs of ageing populations (World Health Organisation 2017). In the UK, Skills for Care set out a *Workforce Strategy for Adult Social Care in England* (Skills for Care 2024), which calls for pay and wellbeing reforms to attract and retain care staff. Also proposed are new standards for competencies attainment to ensure learning and skills development continue to promote the delivery of person‐centred, safe and effective care. In addition, the CQC launched its strategy for modernising service quality assessment procedures. This embraces PCC principles by inviting involvement from a range of actors, including people using care services, family members and care staff (Care Quality Commission 2020; Care Quality Commission 2024b). Continued, whole‐system assessments of the impacts reforming measures have on care stakeholders will be crucial for securing the delivery of high‐quality care and for supporting older adults to live flourishing lives in all care settings.

The content analysis also displayed how participants reported fewer examples of how care planning was related to the *care relationships* domain, compared to the other domains of the Wilberforce et al. (2017) framework. This could be attributed to the fundamental role of planning care, which involves scheduling and organising available resources around residents' needs and preferences. The *care relationships* domain on the other hand, refers to staff skills and provider ideology that underlie the care planning process, rather than a part of the process itself; a position upheld by earlier studies exhibiting how the quality of resident‐staff relationships and communication affect staffs' depth of insight into residents' personhood and in turn, residents' engagement in shared decision‐making and care planning (Bennett et al. 2020; Brown Wilson et al. 2013; Backman et al. 2020).

Nevertheless, almost a third of participants suggested that PCCP approaches could support the coordination and continuity of care, as tools advocating for residents' unique needs and goals across disciplinary teams and with external actors. In addition, CFIR *outer setting* findings confirmed that garnering care plan contributions from a wide range of relevant stakeholders was invaluable for capturing residents' identity and personal care goals. Preceding observations also described the positive associations between PCC practices and care homes cultivating constructive relationships with family members and external care providers (Abbott et al. 2016; Kang et al. 2020; Jobe 2022; Lood et al. 2020). Amid the backdrop of a highly fragmented care system in England of disjointed working relationships between (predominantly) private care home providers and public health care services, which propagates care inequities and fails to support people with highly complex needs over the long term (Szczepura et al. 2023; Care Quality Commission 2024c), our findings perhaps signal broad accord for moving towards more integrated forms of working across health, social and voluntary care sectors to ensure efficient care and preserve dignity in later life (Department of Health and Social Care 2021; World Health Organisation 2017; Curry et al. 2022; Rudnicka et al. 2020).

As the global population continues to age, addressing the challenges facing the long‐term care workforce is a leading public health priority (World Health Organisation 2017). Increasingly, governments are considering the potential for digital technologies to mitigate the growing imbalances between the demand for services and supply of care resources (Hamblin 2022). *Inner setting* domain findings, however, exposed some of the challenges associated with modernisation initiatives that involve the digitalising elements of care practices. Some practitioners expressed optimism for the time and space efficiencies of implementing digital care tools in the planning process, which could redirect care resources towards the interpersonal aspects of resident care. Others suggested that digital planning systems eased access to resident data, which in turn could relieve some of the pressures of staffing shortages. Comparable optimism for the benefits of digital planning tools in the care sector has been recorded elsewhere (Kim et al. 2021; Roberts et al. 2023). However, others found that the (often) nascent digital planning tools failed to capture the humane aspects of care planning. Shiells et al. (2020) similarly reported that electronic care platforms lacked customisability, inhibited the documentation of residents' specific needs, and expressed concerns about the intrusiveness of the technology and consequent risk of dehumanising care delivery. Ausserhofer et al. (2021) noted that time saved by quick access to relevant resident information was offset by a lack of computing equipment for care staff to implement timely care planning. Supported by previous research, our findings emphasise the opportunity for care home nursing practitioners to lead the co‐development of integrated digital planning platforms, in collaboration with colleagues from across the care sector, technology developers and policy makers, that align with person‐centred principles, amplify the voices of frontline nursing and care staff, as well as respond to the needs of contemporary long‐term care settings for older adults (Kemp et al. 2024; Johnston et al. 2022).

### 7.2 Implications for Future Research

Overall, we found there was general appreciation of PCC principles amongst practitioners when planning care, particularly with respect to the Wilberforce et al. (2017) domains of *understanding residents' identity* and *engaging residents in decision‐making*. The PCC construct around staff's understanding of resident's values and preferences in care was most frequently discussed. There were also regular references to the related constructs of involving residents– and incorporating their wishes*—*in the planning process. This may be partly explained by a growing recognition within care policy and regulatory discourse of the benefits of fostering a profound understanding of residents and involving them in planning their own care—an awareness that has filtered through to care professionals and society at large (Lepore et al. 2018). Alternatively, participants' focus on residents' preferences may stem from a tendency amongst care providers to explicate most staff‐resident interactions as person‐centred, rather than a professional commitment to PCC principles (Brooker 2003). Indeed, the CFIR *intervention domain* analysis revealed ambivalence towards PCC practices. These practices were described as both fundamental for delivering high quality residential care and time consuming, impractical and obstructive to other care activities. Participants' reflections on the *outer setting* domain—which concerned regulatory and care practice misalignment and adverse staffing conditions—also suggest that a cursory approach to PCCP might be a coping mechanism, as staff sought to balance regulatory standards with substantial workloads. The links between sustained heavy workload, staff burnout and the depersonalisation of care have been extensively documented (Forsgren and Saldert 2022; Lepore et al. 2018; Richter et al. 2022) and have prompted widespread concerns about the sustainability of current models of long‐term care for older adults (Department of Health and Social Care 2021; Skills for Care 2024). Extant care home workload pressures also point to a need for further grounded research to unpack the meaning of person‐centredness for care home residents and staff, to achieve a deeper understanding of which aspects of PCC are practicable.

### 7.3 Limitations

There are several limitations to this study. Firstly, the research participants were predominantly of a white ethnicity and held managerial positions. Care plans are informed by the daily observations and interactions of—and chiefly implemented by– front‐line care staff, a significant proportion of whom are of a minority ethnic background (Skills for Care, 2024). Their insights on the accessibility and usability of care plans are essential for the continued proliferation of PCC approaches. This points to a need for devising strategies that promote the inclusion of front‐line carers as central contributors to care home research to obtain a full grasp of the salient issues and challenges of the sector.

Moreover, the current analysis does not demonstrate the range of approaches participants took to planning, nor are our findings necessarily reflections of commonly held views or experiences of care planning practice across England. For example, we did not seek to describe examples of more institutional or pathological approaches to planning. Our analysis, therefore, may suggest that person‐centred approaches are commonplace and comprehensive, whereas in fact, a range of care planning approaches were discussed and examples of PCCP were often isolated to a specific domain or facet; adoption of the full PCCP framework by a singular provider cannot be assumed.

Finally, the main purpose of the consultations was to understand how care planning took place, the content and uses of care planning. There was not a specific focus on PCCP in the main study's objectives and interview schedule; participants were not explicitly prompted to reflect on issues relating to person‐centred care planning. Therefore, findings do not adequately represent the extent to which the different aspects of PCCP are understood or take place in English care homes more generally. Moreover, comments about the barriers and facilitators to implementing PCCP are incidental. As such, discussions of the influencing factors of implementation are incomplete and require further exploration.

## 8 Conclusion

Care home practitioners provided examples of PCC planning in terms of understanding residents' goals, values and preferences and engaging residents in decisions about their care. Participants also commented on the value of coordinating care across sectors, through working partnerships with family members and external care services, further emphasising the need for investment into—and investigation of—integrated care systems. Overall, there are enduing differences in care practitioners' understanding and practice of PCCP, which require comprehensive examination for policymakers and care services regulators to arrive at a deeper understanding of the operational capabilities of the care home sector.

The challenges faced by the care home sector, including increasing demand coupled with critical staffing shortages, necessitate cross‐sector, co‐developed solutions that promote time, labour and cost efficiencies, ensure the integrity of care services and promote the dignity and personal preferences of older people with care needs.

## Author Contributions

All authors have agreed on the final version and meet at least one of the following criteria (recommended by the ICMJE: http://www.icmje.org/recommendations/). (1) substantial contributions to conception and design, acquisition of data, or analysis and interpretation of data; (2) drafting the article or revising it critically for important intellectual content.

## Conflicts of Interest

The authors declare no conflicts of interest.

BOX 1 **Person‐centred care framework.**

**Table:** 

Dimensions	Constructs
Understanding the person	Knows different dimensions of life requiring support
	Understanding personal experience of illness and limitations
	Knows what is important to person's identity and wellbeing
	Understands person's values and preferences in care
Engagement in Decision‐making	Person involved in decision‐making process
	Person's wishes shape decisions and care plans
	Flexible care tailored to individual preferences
	Information and options shared in clear format
Promoting care relationships	Friendly, caring, respectful interactions
	Continuity and coordination in care relationships
	Positive attitude to person's capabilities and roles
	Reciprocity in care relationships

*Source*: Wilberforce et al., 2017.

## Supporting information


Appendix S1.

Appendix S2.

## Data Availability

Research data are not shared.

## References

[jan16965-bib-0001] Abbott, K. M. , A. R. Heid , and K. Van Haitsma . 2016. “‘We Can't Provide Season Tickets to the Opera’: Staff Perceptions of Providing Preference Based Person Centered Care.” Clinical Gerontologist 39, no. 3: 190–209. 27134341 10.1080/07317115.2016.1151968 PMC4847948

[jan16965-bib-0002] Ausserhofer, D. , L. Favez , M. Simon , and F. Zuniga . 2021. “Electronic Health Record Use in Swiss Nursing Homes and Its Association With Implicit Rationing of Nursing Care Documentation: Multicenter Cross‐Sectional Survey Study.” JMIR Medical Informatics 9, no. 3: e22974. 33650983 10.2196/22974 PMC7967228

[jan16965-bib-0003] Backman, A. , P. Ahnlund , K. Sjogren , H. Lovheim , K. S. McGilton , and D. Edvardsson . 2020. “Embodying Person‐Centred Being and Doing: Leading Towards Person‐Centred Care in Nursing Homes as Narrated by Managers.” Journal of Clinical Nursing 29, no. 1–2: 172–183. 31612556 10.1111/jocn.15075

[jan16965-bib-0004] Backman, A. , P.‐O. Sandman , and A. Sköldunger . 2021. “Characteristics of Nursing Home Units With High Versus Low Levels of Person‐Centred Care in Relation to Leadership, Staffresident‐ and Facility Factors: Findings From SWENIS, a Cross‐Sectional Study in Sweden.” BMC Geriatrics 21: 1–11. 34530734 10.1186/s12877-021-02434-0 PMC8447583

[jan16965-bib-0005] Ballard, C. , A. Corbett , M. Orrell , et al. 2018. “Impact of Person‐Centred Care Training and Person‐Centred Activities on Quality of Life, Agitation, and Antipsychotic Use in People With Dementia Living in Nursing Homes: A Cluster‐Randomised Controlled Trial.” PLoS Medicine 15, no. 2: e1002500. 29408901 10.1371/journal.pmed.1002500 PMC5800565

[jan16965-bib-0006] Baron, K. , A. Hodgson , and C. Walshe . 2015. “Evaluation of an Advance Care Planning Education Programme for Nursing Homes: A Longitudinal Study.” Nurse Education Today 35, no. 5: 689–695. 25638279 10.1016/j.nedt.2015.01.005

[jan16965-bib-0007] Bavelaar, L. , M. Visser , C. Walshe , et al. 2023. “The Impact of the mySupport Advance Care Planning Intervention on Family Caregivers' Perceptions of Decision‐Making and Care for Nursing Home Residents With Dementia: Pretest‐Posttest Study in Six Countries.” Age and Ageing 52, no. 3: afad027. 36861181 10.1093/ageing/afad027 PMC9978311

[jan16965-bib-0008] Bengtsson, M. 2016. “How to Plan and Perform a Qualitative Study Using Content Analysis.” NursingPlus Open 2: 8–14. 10.1016/j.npls.2016.01.001.

[jan16965-bib-0009] Bennett, M. , K. von Treuer , M. P. McCabe , et al. 2020. “Resident Perceptions of Opportunity for Communication and Contribution to Care Planning in Residential Aged Care.” International Journal of Older People Nursing 15, no. 1: e12276. 10.1111/opn.12276. 31578823

[jan16965-bib-0010] Berg, V. 2025. “Care Home Facts, Stats. Settings, Population and WORKFORCE: carehome.co.uk.” Accessed January 31, 2025. https://www.carehome.co.uk/advice/care‐home‐stats‐number‐of‐settings‐population‐workforce.

[jan16965-bib-0011] Blake, D. , K. Berry , and L. J. E. Brown . 2020. “A Systematic Review of the Impact of Person‐Centred Care Interventions on the Behaviour of Staff Working in Dementia Care.” Journal of Advanced Nursing 76: 426–444. 31657034 10.1111/jan.14251

[jan16965-bib-0012] Brooker, D. 2003. “What Is Person‐Centred Care in Dementia?” Reviews in Clinical Gerontology 13, no. 3: 215–222.

[jan16965-bib-0013] Brown Wilson, C. , C. Swarbrick , M. Pilling , and J. Keady . 2013. “The Senses in Practice: Enhancing the Quality of Care for Residents With Dementia in Care Homes.” Journal of Advanced Nursing 69, no. 1: 77–90. 22462405 10.1111/j.1365-2648.2012.05992.x

[jan16965-bib-0014] Care Quality Commission . 2020. “A New Strategy for the Changing World of Health and Social Care: Our Strategy From 2021.” Newcastle Upon Tyne; Contract No.: CQC‐471‐052021.

[jan16965-bib-0015] Care Quality Commission . 2024a. “Assessment Framework: Care Quality Commission.” Accessed February 22, 2024. https://www.cqc.org.uk/guidance‐regulation/providers/assessment/single‐assessment‐framework/responsive/person‐centred‐care.

[jan16965-bib-0016] Care Quality Commission . 2024b. “Assessing Quality and Performance: Care Quality Commission.” Accessed May 21, 2024; October 02, 2024. https://www.cqc.org.uk/guidance‐regulation/providers/assessment/assessing‐quality‐and‐performance.

[jan16965-bib-0017] Care Quality Commission . 2024c. “The State of Health Care and Adult Social Care in England 2023/24.”

[jan16965-bib-0018] Chenoweth, L. , I. Forbes , R. Fleming , et al. 2014. “PerCEN: A Cluster Randomized Controlled Trial of Person‐Centered Residential Care and Environment for People With Dementia.” International Psychogeriatrics 26, no. 7: 1147–1160. 24666667 10.1017/S1041610214000398

[jan16965-bib-0019] Coleman, J. , J. Levy , S. Wiggins , and J. Kinley . 2017. “Using a New End‐Of‐Life Care Plan in Nursing Homes.” Nursing and Residential Care 19, no. 1: 38–41.

[jan16965-bib-0020] Cooper, C. , L. Marston , J. Barber , et al. 2018. “Do Care Homes Deliver Person‐Centred Care? A Cross‐Sectional Survey of Staff‐Reported Abusive and Positive Behaviours Towards Residents From the MARQUE (Managing Agitation and Raising Quality of Life) English National Care Home Survey.” PLoS One 13, no. 3: e0193399. 29561867 10.1371/journal.pone.0193399 PMC5862450

[jan16965-bib-0021] Coulourides Kogan, A. , K. Wilber , and L. Mosqueda . 2016. “Moving Toward Implementation of Person‐Centered Care for Older Adults in Community‐Based Medical and Social Service Settings: “You Only Get Things Done When Working in Concert With Clients”.” Journal of the American Geriatrics Society 64: e8–e14. 26626544 10.1111/jgs.13876

[jan16965-bib-0022] Curry, L. , A. Ayedun , E. Cherlin , B. Taylor , S. Castle‐Clarke , and E. Linnander . 2022. “The Role of Leadership in Times of Systems Disruption: A Qualitative Study of Health and Social Care Integration.” BMJ Open 12, no. 5: e054847. 10.1136/bmjopen-2021-054847 PMC9108434 35568492

[jan16965-bib-0023] Damschroder, L. J. , D. C. Aron , R. E. Keith , S. R. Kirsh , J. A. Alexander , and J. C. Lowery . 2009. “Fostering Implementation of Health Services Research Findings Into Practice: A Consolidated Framework for Advancing Implementation Science.” Implementation Science 4: 50. 19664226 10.1186/1748-5908-4-50 PMC2736161

[jan16965-bib-0024] Damschroder, L. J. , C. M. Reardon , M. A. O. Widerquist , and J. Lowery . 2022. “The Updated Consolidated Framework for Implementation Research Based on User Feedback.” Implementation Science 17, no. 1: 75. 36309746 10.1186/s13012-022-01245-0 PMC9617234

[jan16965-bib-0025] de Silva, D. 2014. “Helping Measure Person‐Centred Care: A Review of Evidence About Commonly Use Approaches and Tools Used to Healp Measure Person‐Centred Care” London.

[jan16965-bib-0026] Department of Health and Social Care . 2001. “National Service Framework for Older People.” London.

[jan16965-bib-0027] Department of Health and Social Care . 2014. “Care and Support Statutory Guidance: Issued Under the Care Act 2014.” London.

[jan16965-bib-0028] Department of Health and Social Care . 2021. “People at the Heart of Care: Adult Social Care Reform White Paper.” London; Contract No.: CP 560.

[jan16965-bib-0029] Dobie, J. , M. Plumb , and S. Shephard . 2016. “End of Life Care Education to Care Home Staff: An Evaluation.” Nursing and Residential Care 18, no. 7: 369–374.

[jan16965-bib-0030] Erkes, J. , C. J. Camp , and S. Bayard . 2022. “Don't Bother Trying, They Won't Do It! Effect of Responsive Behaviors on the Montessori Assessment System.” Clinical Gerontologist 45, no. 4: 870–877. 33998979 10.1080/07317115.2021.1924333

[jan16965-bib-0031] Farrington, C. J. 2014. “Blended e‐Learning and End of Life Care in Nursing Homes: A Small‐Scale Mixed‐Methods Case Study.” BMC Palliative Care 13: 31. 24994948 10.1186/1472-684X-13-31 PMC4080686

[jan16965-bib-0032] Finucane, A. M. , B. Stevenson , R. Moyes , D. Oxenham , and S. A. Murray . 2013. “Improving End‐Of‐Life Care in Nursing Homes: Implementation and Evaluation of an Intervention to Sustain Quality of Care.” Palliative Medicine 27, no. 8: 772–778. 23612957 10.1177/0269216313480549

[jan16965-bib-0033] Forsgren, E. , and C. Saldert . 2022. “Implementation of Communication Routines Facilitating Person‐Centred Care in Long‐Term Residential Care: A Pilot Study.” Health Expectations 25, no. 6: 2982–2991. 36148650 10.1111/hex.13606 PMC9700177

[jan16965-bib-0034] Fossey, J. , S. Masson , J. Stafford , V. Lawrence , A. Corbett , and C. Ballard . 2014. “The Disconnect Between Evidence and Practice: A Systematic Review of Person‐Centred Interventions and Training Manuals for Care Home Staff Working With People With Dementia.” International Journal of Geriatric Psychiatry 29, no. 8: 797–807. 24535885 10.1002/gps.4072

[jan16965-bib-0035] Garden, G. , A. Usman , D. Readman , et al. 2022. “Advance Care Plans in UK Care Home Residents: A Service Evaluation Using a Stepped Wedge Design.” Age and Ageing 51, no. 3: afac069. 35348604 10.1093/ageing/afac069 PMC8963445

[jan16965-bib-0036] Gilissen, J. , A. Wendrich‐van Dael , C. Gastmans , et al. 2021. “Differences in Advance Care Planning Among Nursing Home Care Staff.” Nursing Ethics 28, no. 7–8: 1210–1227. 33947293 10.1177/0969733021994187

[jan16965-bib-0037] Guney, S. , A. Karadag , and M. El‐Masri . 2021. “Perceptions and Experiences of Person‐Centered Care Among Nurses and Nurse Aides in Long Term Residential Care Facilities: A Systematic Review of Qualitative Studies.” Geriatric Nursing 42, no. 4: 816–824. 34090225 10.1016/j.gerinurse.2021.04.005

[jan16965-bib-0038] Gustavsson, K. , C. van Diepen , A. Fors , M. Axelsson , M. Bertilsson , and G. Hensing . 2023. “Healthcare Professionals' Experiences of Job Satisfaction When Providing Person‐Centred Care: A Systematic Review of Qualitative Studies.” BMJ Open 13, no. 6: e071178. 10.1136/bmjopen-2022-071178 PMC10277035 37295826

[jan16965-bib-0039] Hamblin, K. 2022. “Sustainable Social Care: The Potential of Mainstream “Smart” Technologies.” Sustainability 14, no. 5: 21.

[jan16965-bib-0040] Heckman, G. A. , V. Boscart , P. Quail , et al. 2022. “Applying the Knowledge‐To‐Action Framework to Engage Stakeholders and Solve Shared Challenges With Person‐Centered Advance Care Planning in Long‐Term Care Homes.” Canadian Journal on Aging 41, no. 1: 110–120. 33583447 10.1017/S0714980820000410

[jan16965-bib-0041] Jobe, I. 2022. “Reflections of the Collaborative Care Planning as a Person‐Centred Practice.” Nursing Philosophy 23, no. 3: e12389. 35322917 10.1111/nup.12389 PMC9285900

[jan16965-bib-0042] Johnston, L. , H. Koikkalainen , L. Anderson , P. Lapok , A. Lawson , and S. D. Shenkin . 2022. “Foundation Level Barriers to the Widespread Adoption of Digital Solutions by Care Homes: Insights From Three Scottish Studies.” International Journal of Environmental Research and Public Health 19, no. 12: 7407. 35742667 10.3390/ijerph19127407 PMC9223833

[jan16965-bib-0043] Kang, B. , K. Scales , E. S. McConnell , Y. Song , M. Lepore , and K. Corazzini . 2020. “Nursing Home Residents' Perspectives on Their Social Relationships.” Journal of Clinical Nursing 29, no. 7–8: 1162–1174. 31889360 10.1111/jocn.15174 PMC7085465

[jan16965-bib-0044] Kemp, D. , M. Doyle , M. Turner , and S. Hemingway . 2024. “Care Plan Templates in Adult Community Mental Health Teams in England and Wales: An Evaluation.” Nursing Reports 14, no. 1: 340–352. 38391071 10.3390/nursrep14010026 PMC10885041

[jan16965-bib-0045] Kesten, J. M. , S. Redwood , A. Pullyblank , et al. 2022. “Using the Recommended Summary Plan for Emergency Care and Treatment (ReSPECT) in Care Homes: A Qualitative Interview Study.” Age and Ageing 51, no. 10: afac226. 36273344 10.1093/ageing/afac226 PMC9588387

[jan16965-bib-0046] Kim, H. , Y. I. Jung , G. S. Kim , H. Choi , and Y. H. Park . 2021. “Effectiveness of a Technology‐Enhanced Integrated Care Model for Frail Older People: A Stepped‐Wedge Cluster Randomized Trial in Nursing Homes.” Gerontologist 61, no. 3: 460–469. 32668005 10.1093/geront/gnaa090 PMC8355475

[jan16965-bib-0047] Kitwood, T. , and K. Bredin . 1992. “Towards a Theory of Dementia Care: Personhood and Well‐Being.” Ageing and Society 12: 269–287. 11654434 10.1017/s0144686x0000502x

[jan16965-bib-0048] Lepore, M. , K. Scales , R. A. Anderson , et al. 2018. “Person‐Directed Care Planning in Nursing Homes: A Scoping Review.” International Journal of Older People Nursing 13, no. 4: e12212. 30358099 10.1111/opn.12212 PMC6282715

[jan16965-bib-0049] Lood, Q. , K. Sjogren , A. Bergland , et al. 2020. “Effects of a Staff Education Programme About Person‐Centred Care and Promotion of Thriving on Relatives' Satisfaction With Quality of Care in Nursing Homes: A Multi‐Centre, Non‐Equivalent Controlled Before‐After Trial.” BMC Geriatrics 20, no. 1: 268. 32738880 10.1186/s12877-020-01677-7 PMC7395407

[jan16965-bib-0050] Manthorpe, J. , and K. Samsi . 2016. “Person‐Centered Dementia Care: Current Perspectives.” Clinical Interventions in Aging 11: 1733–1740. 27932869 10.2147/CIA.S104618 PMC5135058

[jan16965-bib-0051] Moyle, W. , D. Parker , and M. Bramble . 2014. Care of Older Adults: A Strengths‐Based Approach. Cambridge University Press xv, 213 pages.

[jan16965-bib-0052] National Institue for Health and Care Excellence . 2015a. Home Care: Delivering Personal Care and Practical Support to Older People Living in Their Own Homes, 32. National Institute for Health and Care Excellence.

[jan16965-bib-0053] National Institue for Health and Care Excellence . 2015b. Transition Between Inpatient Hospital Settings and Community or Care Home Settings for Adults With Social Care Needs, 33. National Institute for Health and Care Excellence.

[jan16965-bib-0054] National Institue for Health and Care Excellence . 2024. “NICEimpact Dementia: Social Care.” NICE; 2024 Accesed November 06, 2024. https://www.nice.org.uk/about/what‐we‐do/into‐practice/measuring‐the‐use‐of‐nice‐guidance/impact‐of‐our‐guidance/niceimpact‐dementia/ch4‐social‐care#:~:text=People%27s%20experience%20of%20care%20and,about%20their%20care%20and%20support.

[jan16965-bib-0055] NIHR Applied Research Collaboration Kent SaS . 2020. “Well‐Being in Care Homes: Implementation of (Outcomes‐Based) Care Planning: National Institute for Health and Care Research.” https://arc‐kss.nihr.ac.uk/npp‐adult‐social‐care‐social‐work/our‐programme‐of‐work/well‐being‐in‐care‐homes‐implementation‐of‐outcomes‐based‐care‐planning.

[jan16965-bib-0057] O'Brien, M. , J. Kirton , K. Knighting , B. Roe , and B. Jack . 2016. “Improving End of Life Care in Care Homes; an Evaluation of the Six Steps to Success Programme.” BMC Palliative Care 15: 53. 27259551 10.1186/s12904-016-0123-6 PMC4891879

[jan16965-bib-0058] Office for National Statistics . 2023. “Older People Living in Care Homes and Changes Over Time, England and Wales, Census 2021.” London.

[jan16965-bib-0059] Richter, C. , S. Fleischer , H. Langner , et al. 2022. “Factors Influencing the Implementation of Person‐Centred Care in Nursing Homes by Practice Development Champions: A Qualitative Process Evaluation of a Cluster‐Randomised Controlled Trial (EPCentCare) Using Normalization Process Theory.” BMC Nursing 21, no. 1: 182. 35804407 10.1186/s12912-022-00963-6 PMC9264574

[jan16965-bib-0060] Ritchie, J. , and J. Lewis . 2003. Qualitative Research Practice: A Guide for Social Science Students and Researchers. Sage Publications xv, 336 pp.

[jan16965-bib-0061] Roberts, R. L. , D. P. Mohan , K. D. Cherry , et al. 2023. “Deployment of a Digital Advance Care Planning Platform at an Accountable Care Organization.” Journal of the American Board of Family Medicine 36, no. 6: 966–975. 10.3122/jabfm.2023.230133r2. 37907349

[jan16965-bib-0062] Rudnicka, E. , P. Napierala , A. Podfigurna , B. Meczekalski , R. Smolarczyk , and M. Grymowicz . 2020. “The World Health Organization (WHO) Approach to Healthy Ageing.” Maturitas 139: 6–11. 32747042 10.1016/j.maturitas.2020.05.018 PMC7250103

[jan16965-bib-0063] Shiells, K. , A. A. Diaz Baquero , O. Stepankova , and I. Holmerova . 2020. “Staff Perspectives on the Usability of Electronic Patient Records for Planning and Delivering Dementia Care in Nursing Homes: A Multiple Case Study.” BMC Medical Informatics and Decision Making 20, no. 1: 159. 32660474 10.1186/s12911-020-01160-8 PMC7359585

[jan16965-bib-0064] Skills for Care . 2024. “The State of the Adult Social Care Sector and Workforce in England: 2024.” Leeds.

[jan16965-bib-0065] Smith, N. , J. Damant , Y. Hamashima , et al. 2024. “Care Planning in Older Adult Care Homes: A Qualitative Study of Care Staff Experiences and Views.” International Journal of Care and Caring: 1–18. 10.1332/23978821Y2024D000000092..

[jan16965-bib-0066] Social Care Institute for Excellence . 2017. Person‐Centred Care for Older People in Care Homes. Social Care Institute for Excellence.

[jan16965-bib-0067] Social Care Institute for Excellence . 2025. “Care Homes as a Model for Housing With Care and Support: Social Care Institute for Excellence.” https://www.scie.org.uk/housing/role‐of‐housing/promising‐practice/models/care‐home/.

[jan16965-bib-0068] Sopcheck, J. , and R. M. Tappen . 2023. “Nursing Home Resident, Family, and Staff Perspectives on Hospital Transfers for End‐Of‐Life Care.” Omega (Westport) 86, no. 3: 1046–1068. 33632028 10.1177/0030222821997708

[jan16965-bib-0069] Spacey, A. , J. Scammell , M. Board , and S. Porter . 2021. “A Critical Realist Evaluation of Advance Care Planning in Care Homes.” Journal of Advanced Nursing 77, no. 6: 2774–2784. 33751625 10.1111/jan.14822

[jan16965-bib-0070] Steel, A. , H. Hopwood , E. Goodwin , and E. L. Sampson . 2022. “Multidisciplinary Residential Home Intervention to Improve Outcomes for Frail Residents.” BMC Health Services Research 22, no. 1: 58. 35022056 10.1186/s12913-021-07407-y PMC8756619

[jan16965-bib-0071] Stevens, E. , S. G. Clarke , J. Harrington , et al. 2022. “The Provision of Person‐Centred Care for Care Home Residents With Stroke: An Ethnographic Study.” Health & Social Care in the Community 30, no. 6: e5186–e5195. 10.1111/hsc.13936. 35869786 PMC10084099

[jan16965-bib-0072] Stone, L. , J. Kinley , and J. Hockley . 2013. “Advance Care Planning in Care Homes: The Experience of Staff, Residents, and Family Members.” International Journal of Palliative Nursing 19, no. 11: 550–557. 24263899 10.12968/ijpn.2013.19.11.550

[jan16965-bib-0073] Sussman, T. , J. Lawrence , A. Earn , M. Wilkie , P. Hunter , and S. Kaasalainen . 2023. “Chart Based Data as a Resource for Tracking and Improving a Person‐Centred Palliative Approach in Long‐Term Care.” Journal of Clinical Nursing 32, no. 13–14: 4049–4059. 36225135 10.1111/jocn.16558

[jan16965-bib-0074] Szczepura, A. , H. Masaki , D. Wild , T. Nomura , M. Collinson , and R. Kneafsey . 2023. “Integrated Long‐Term Care ‘Neighbourhoods’ to Support Older Populations: Evolving Strategies in Japan and England.” International Journal of Environmental Research and Public Health 20, no. 14: 6352. 37510584 10.3390/ijerph20146352 PMC10379849

[jan16965-bib-0075] Taylor, J. , N. Smith , L. Prato , et al. 2023. “Care Planning Interventions for Care Home Residents: A Scoping Review.” Journal of Long‐Term Care 2023: 326–337.

[jan16965-bib-0076] Van den Block, L. , E. Honinx , L. Pivodic , et al. 2019. “Evaluation of a Palliative Care Program for Nursing Homes in 7 Countries: The PACE Cluster‐Randomized Clinical Trial.” JAMA Internal Medicine 180, no. 2: 233–242. 10.1001/jamainternmed.2019.5349 PMC6865772 31710345

[jan16965-bib-0077] van den Pol‐Grevelink, A. , J. S. Jukema , and C. H. Smits . 2012. “Person‐Centred Care and Job Satisfaction of Caregivers in Nursing Homes: A Systematic Review of the Impact of Different Forms of Person‐Centred Care on Various Dimensions of Job Satisfaction.” International Journal of Geriatric Psychiatry 27, no. 3: 219–229. 21538536 10.1002/gps.2719

[jan16965-bib-0078] Wickson‐Griffiths, A. , S. Kaasalainen , J. Ploeg , and C. McAiney . 2014. “A Review of Advance Care Planning Programs in Long‐Term Care Homes: Are They Dementia Friendly?” Nursing Research & Practice 2014: 875897. 24757563 10.1155/2014/875897 PMC3976775

[jan16965-bib-0079] Wilberforce, M. , D. Challis , L. Davies , M. P. Kelly , C. Roberts , and P. Clarkson . 2017. “Person‐Centredness in the Community Care of Older People: A Literature‐Based Concept Synthesis.” International Journal of Social Welfare 26: 86–98.

[jan16965-bib-0080] World Health Organisation . 2017. “Global Strategy and Action Plan on Ageing and Health.” Switzerland.

