# Supplementary figures and images for: Investigating Person‐Centred Care Planning in Care Homes Across England: An Exploratory Study of Practices and Contextual Factors
Source: J Adv Nurs. 2025 Apr 7;82(1):617–31. doi: 10.1111/jan.16965 (PMC12721941; doi:10.1111/jan.16965)

Supplementary File 1: NIHR Applied Research Collaboration National Priority Programme


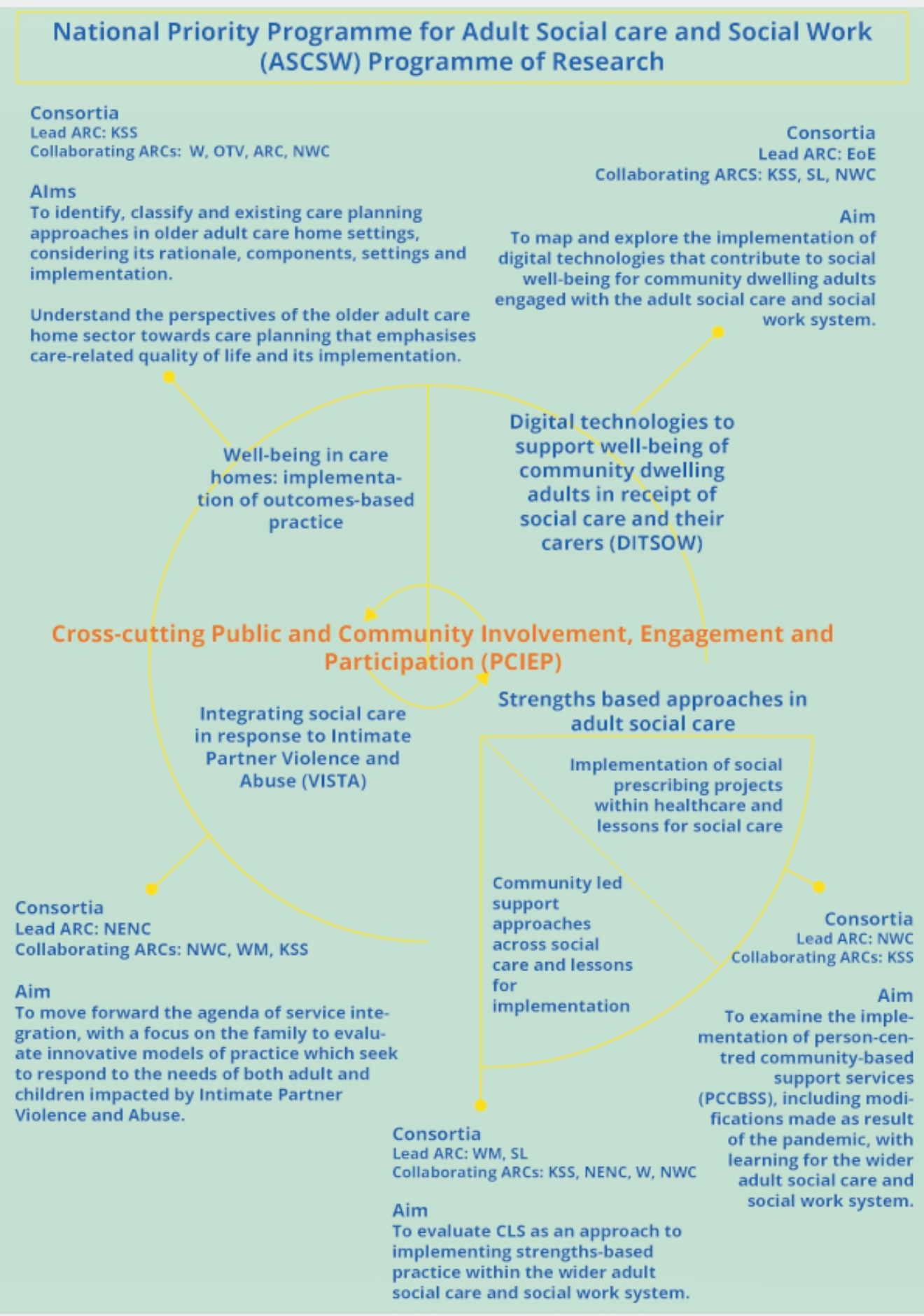

Supplement: Supplementary file 1 — Appendix S1. [file JAN-82-617-s002.docx]
