# Supplementary material for: Investigating Person‐Centred Care Planning in Care Homes Across England: An Exploratory Study of Practices and Contextual Factors
Source: J Adv Nurs. 2025 Apr 7;82(1):617–31. doi: 10.1111/jan.16965 (PMC12721941; doi:10.1111/jan.16965)
Supplement: Supplementary file 2 — Appendix S2. [file JAN-82-617-s001.docx]

# ARC NPP: Wellbeing in care homes

# Consultation interview and topic guide

## Section One: Introduction

### Key points to cover:

- ***What the study/consultation is about:*** This consultation is about improving approaches to care planning in older adult care homes that aim to improve residents’ quality of life. We would like to understand your perspectives and views on how you currently conduct care planning, the challenges you might face undertaking care planning with residents and ways in which it could be improved.
- We also know that making successful changes to routine practice can be difficult, so we would also like to seek your views on the best ways of introducing new ways of working in this setting.
- **How the consultation interview/workshop will work, including:**
  - How long it will last.
  - It is about their views and experiences and therefore there are no wrong answers.
  - They may refuse to answer any questions or stop the interview at any point.
  - Check consent to record.
  - What will happen with the information they share, including:
    - How it will be used: The results of this consultation will be shared in reports, academic journal articles, conference papers, summary findings and other formats (e.g. blogs). If you give your permission, we will send you a summary of the results at the end of the consultation. The information you share will help us in improving approaches to care planning that aims to improve residents’ wellbeing and quality of life.
    - How identifiable they will be: The researchers will not use your name or location in any reports of this work. Reports will not contain any personally identifiable information. The reports may use direct quotes, as well as more general description of the information that you share.
    - How the information will be stored: The information will be stored on secure servers at the University of Kent. We will hold your personal information for the duration of the consultation, so we are able to contact you to arrange an interview, for data audit/verification purposes and also to send you a copy of the findings at the end of the consultation. Your personal information will be stored separately to the audio recording and transcripts of the consultation. This information will only be accessible to the research team at the Universities of Bristol, Kent, Liverpool, Oxford, and the London School of Economics and Political Science. The database with your personal information and audio files will be securely destroyed three months after the study has finished. We will keep copies of the anonymised transcripts indefinitely, for the purposes of data audit and further analysis/reporting. The University of Kent is the Sponsor for this study based in Canterbury, Kent (UK) and the data controller. We will be using information from you in order to undertake this consultation. The consultation will be compliant with the Data Protection Act 2018 and the General Data Protection Regulations with regards to the collection, storage, processing and disclosure of personal information and will uphold the Act’s core principles.

## Section two: Consultation

### Introduction

Prompts:

- Participant’s role in the care home
- What they understand by the term ‘care planning’

### Current care planning processes in care homes.

Following a broad question about how care planning is conducted in the home(s) they work in/with, prompts on the topics below could be used:

- Aim of care planning
- Focus of care planning
- Resident, family member, staff involvement
- Timing of care planning (entering the home, annually, change in circumstance)
- Who has a care plan (everyone, those who struggle to communicate, end of life etc.)
- Organisational support to conduct care planning, including training
- Implementing/delivering the care plan
- Recording and sharing the care plan
- Reviewing care plans

### The use of care planning data.

Following a broad question on how care planning information is used by/in the care home, prompts on the topics below could be used:

- Who uses the care plan/information?
- Use of information/care plan outside the care home/across the health and social care system
- Use of aggregated data from care plans
- Other ways information from the care plan could be used

### Challenges in conducting care planning in care homes.

*(If short of time consider missing the next section on improving care planning. If you do, you could add some questions into this section about addressing the challenges identified by the participant*)

Following a broad question about encountering challenges and difficulties in conducting/support care planning, prompts on the topics below could be used:

- Resources to conduct care planning (time/staff)
- Organisational support, such as training, systems
- Involving residents, family and friends
- Reviewing the care plan.
- Implementing and delivering the care plan

### Improving care planning in care homes.

*(see note above about missing this section out if short of time)*

Following a broad question about what could be done to improve care planning in the participant’s care home, prompts on the following topics could be used:

- Addressing any challenges outlined in the previous section
- Action needed to improve care planning.
- Barriers to improving care planning

### The potential of quality-of-life based care planning. (this section could be missed out if short of time)

Following a broad question asking about what impact care planning can have on a resident’s quality of life, prompts on the topics below could be used:

- The meaning of quality of life
- How care planning can improve wellbeing or quality of life
- Other potential care planning impacts

### Implementing changes in care planning.

Following a broad question about experiencing changes in how care planning is conducted, different sets of prompts could be followed:

If the participant has experience of changes to how care planning is conducted:

- Details of changes
- Impact, success, and sustainability of changes
- factors that facilitated the changes.
- factors that acted as barriers to the changes

If the participant does not have experience of changes to how care planning is conducted, focus on asking them to think hypothetically about the implementation of changes to care planning (if they find this difficult, you could ask them to think about any changes to routine practice in the care home they have experienced):

- factors that might facilitate implementation.
- Factors that might act as barriers to implementation
